# Can sugar metabolism in the cambial region explain the water deficit tolerance in poplar?

**DOI:** 10.1093/jxb/ery195

**Published:** 2018-05-26

**Authors:** Silvia Traversari, Alessandra Francini, Maria Laura Traversi, Giovanni Emiliani, Carlo Sorce, Luca Sebastiani, Alessio Giovannelli

**Affiliations:** 1 Institute of Life Sciences, Scuola Superiore Sant’Anna, Piazza Martiri della Libertà, Pisa, Italy; 2 Trees and Timber Institute (IVALSA-CNR), Via Madonna del Piano, Sesto F.no (Florence), Italy; 3 Department of Biology, University of Pisa, Via Luca Ghini, Pisa, Italy

**Keywords:** Cambial region, carbohydrates, carbon turnover, *Populus*, recovery, starch, water deficit

## Abstract

Drought dramatically affects wood production by adversely impacting cambial cells and their derivatives. Photosynthesis and assimilate transport are also affected by drought conditions. Two poplar genotypes, *Populus deltoides* ‘Dvina’ and *Populus alba* ‘Marte’, demonstrated contrasting growth performance and water–carbon balance strategies; a mechanistic understanding of the water deficit response was provided by these poplar species. ‘Marte’ was found to be more anisohydric than ‘Dvina’. This characteristic was associated with the capacity to reallocate carbohydrates during water deficits. In contrast, ‘Dvina’ displayed more conservative water management; carbohydrates were preferably stored or used for cellulose production rather than to achieve an osmotic balance between the phloem and the xylem. Data confirmed that the more ‘risk-taking’ characteristic of ‘Marte’ allowed a rapid recovery following water deficit and was connected to a different carbohydrate metabolism.

## Teaser

In two poplar species with contrasting growth performance, sugar metabolism in xylem and cambial regions can explain their different grades of tolerance to drought and their recovery rates following water deficits.

## Introduction

Drought dramatically affects the production and yield of plants worldwide, and it is expected to affect plants more frequently due to ongoing global climatic changes (Shao *et al.*, 2008; Osakabe *et al.*, 2014). Water deficits impair the formation of wood directly by decreasing turgor pressure in the cambial cells and their derivatives (Shao *et al.*, 2008; Körner, 2015). Moreover, following the reduction of photosynthesis and assimilate transport, carbon is depleted in the cambial region of plants (Diaz-Espejo and Hernandez-Santana, 2017). Hydraulic failure, carbon starvation, and phloem failure are the principal processes that occur in woody plants under conditions of water deficit, and these result in a decrease in plant growth and an increase in tree mortality (Anderegg *et al.*, 2012). The relationship between transport failure and carbon sink depletion is currently being investigated (Hartmann, 2015), but some evidence has shown that drought tolerance strategies may be based on the modulation of carbohydrate metabolism and osmotic balance (Sevanto *et al.*, 2014). There is a strong relationship between phloem and xylem tissues that ensures a balance between carbon and water (Diaz-Espejo and Hernandez-Santana, 2017). Thus, the relationship between transport failure and carbon sink depletion should be studied carefully by considering the possible integration and co-ordination of phloem and xylem tissues.

The cambial region of a plant includes the cambium, which is composed of meristematic cells called ‘initials’, and mother cells of the xylem and phloem that undergo expansion and differentiation processes (Uggla and Sundberg, 2002). The rate and duration of cambium activity and cell differentiation determine xylem traits, which are crucial for defining the efficiency of water and nutrient transport from roots to shoots (Sorce *et al.*, 2013) as well as the mechanical properties of wood (Lachenbruch and McCulloh, 2014). In this context, useful information can be obtained by studying the metabolic processes that occur in the cambial region during water deficits.

The poplar species can provide a mechanistic understanding of water deficit responses in trees. Within the genus *Populus*, different species show contrasting water-use efficiencies and adaptive strategies under drought conditions (Yin *et al.*, 2004, 2005; Monclus *et al.*, 2009). In poplar species, the sensitivity of the cambial region to water deficit was found to be a genotype-dependent feature; it was related to the activation of different metabolic pathways and contrasting osmotic regulation strategies (Pallara *et al.*, 2012). Water deficit in *Populus deltoides* ‘Dvina’ was found to affect the anatomical properties of the xylem produced following rehydration (Cocozza *et al.*, 2011). In contrast, in *Populus alba* ‘Marte’, the xylem features were unchanged (Luisi *et al.*, 2014). The ability to restore cambium cell division is a key point and allows rapid recovery following rehydration. The crucial checkpoint for mitosis is regulated by B-type cyclins (Fowler *et al.*, 1998), and, in poplar species, gene transcriptions of isoforms *CycB1.3-4* and *CycB2.1* are the highest in dedifferentiated tissues (Emiliani *et al.*, 2016). We hypothesized the role of these genes in the genotype-dependent response of the cambial region to water deficit. Moreover, the analysis of B-type cyclins in the cambial region was combined with the analysis of radial stem variations through point dendrometers. This allowed the verification of the hypothesis proposed by Zweifel *et al.* (2016) that the stem growth of trees is interrupted during times of water deficit (zero growth, the ZG concept).

Many studies have reported the involvement of soluble sugars in response to water deficits (Galvez *et al.*, 2011; Adams *et al.*, 2013; Sengupta and Majumder, 2014; Sevanto *et al.*, 2014; Spicer, 2014; Zwieniecki and Secchi, 2015). Carbohydrates stabilize proteins and membranes, and provide osmotic adjustments and energy reserves for recovery following rehydration. Furthermore, these molecules operate as signal molecules eliciting growth and stress responses (Santos and Pimentel, 2009, Emiliani *et al.*, 2016). Water deficits activate osmotic adjustments by fluctuating non-structural carbon compounds (Blum, 2017), which are in stored and non-stored forms. Although the sucrose/starch pathway has usually been studied by considering only the amount of carbohydrates (Sala *et al.*, 2012; Adams *et al.*, 2013; Lintunen *et al.*, 2016), different genotype-dependent responses could be explained by analysing some of the genes associated with sucrose/starch conversion. In this work, the degradation of sucrose was monitored by gene transcription of *Sus2* and *Sus3* sucrose synthase genes, the two isoforms predominantly expressed in the cambial region of poplar species (Zhang *et al.*, 2011). Interest in the *Sus2* gene has also emerged from its involvement in the biosynthetic pathway of cellulose (Konishi *et al.*, 2004; Coleman *et al.*, 2009) to assess a possible investment in cellulose production, as previously suggested in *P. deltoides* during periods of water deficit (Cocozza *et al.*, 2011). The synthesis and degradation of starch were monitored by the gene transcription of starch synthases (*SS*) and α- and β-amylases (*Amy* and *Bam*). Starch synthases were found to elicit a drought response in poplar species (Street *et al.*, 2006), and *SS2* and *SS4* in *Arabidopsis thaliana* seemed to be involved in different functions, such as starch chain elongation and starch granule initiation, respectively (Streb and Zeeman, 2012). In poplar species, osmotic stress induces the expression of α-amylase genes, such as *Amy1.1-2* (Bae *et al.*, 2010). Furthermore, β-amylase genes, such as *Bam5*, were involved in the refilling of embolized vessels (Secchi and Zwieniecki, 2011). Specifically, *Bam5* was transcriptionally induced by sugars and was the only β-amylase with cytosolic localization (Monroe *et al.*, 2014). Carbohydrate transport was able to be investigated by the transcription of sucrose transporter genes, such as *Suc2.1*, *SUT2.a-b*, and *SUT4*, for their role in drought response in the xylem of poplar (Secchi and Zwieniecki, 2011).

In this context, we performed an experiment to validate the mechanistic hypothesis that genotype-dependent tolerance to water deficit could be explained by regulating specific metabolic pathways related to sugar metabolism in the cambial region of poplar species. Specifically, our hypotheses were as follows: (i) poplar species showing rapid recovery following water deficit exhibit the greatest plasticity in the sucrose/starch pathway; (ii) carbohydrate metabolism of the cambial region is associated with different genotypic responses under water deficit conditions; and (iii) genes related to the cell cycle and sucrose/starch pathway elucidate different degrees of poplar tolerance to water deficit.

## Materials and methods

### Plant material and treatment

**Fig. 1.**
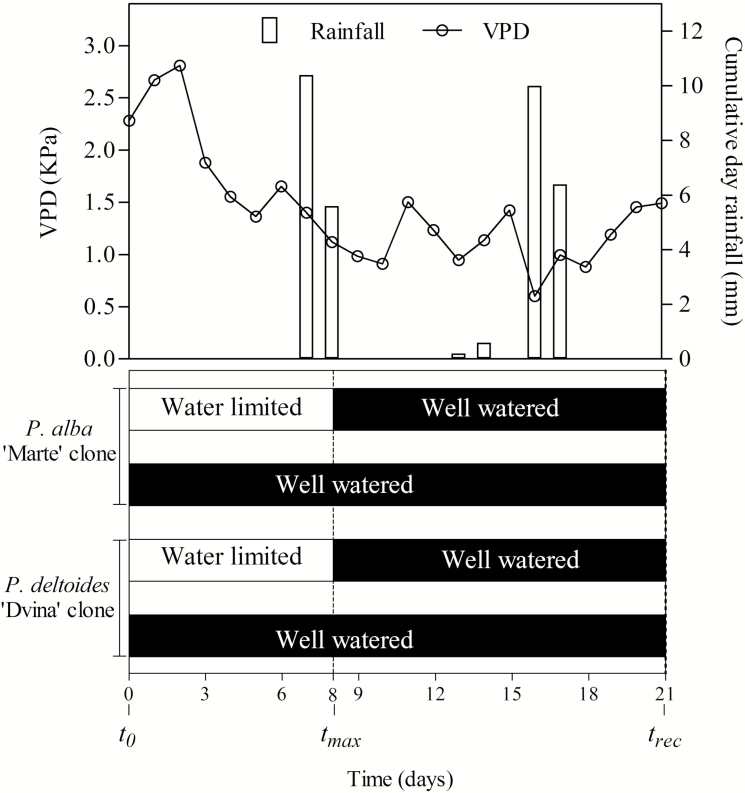
Diagram of 21 d of the experimental plane with the *P. deltoides* ‘Dvina’ clone and the *P. alba* ‘Marte’ clone. Plant samplings were made at *t*_0_ (0 d, *n*=3), after 8 d of withholding water (*t*_max_, *n*=4), and 13 d after resumption of irrigation (*t*_rec_, *n*=4). Meteorological data are reported in the figure.

One-year-old plants of *Populus deltoides* Marsh ‘Dvina’ and *Populus alba* L. ‘Marte’ clones were grown in pots with a combination of peat, sand, and perlite (50/40/10 v/v/v, pH 6.8) at a nursery of CNR-IVALSA in Florence (43°49'06''N 11°12'08''E), as described by Luisi *et al.* (2014). For each genotype, 20 plants were selected for dimensional uniformity. On 12 July (*t*_0_), three plants of each genotype were sampled, and the remaining plants were subjected to different watering regimes (*n*=4) during 21 d of treatment. Soil moisture was kept at field capacity [water content was 30% (w/w) of total soil weight] in well-watered (WW) plants. Watering was suspended for 8 d (*t*_max_) and then resumed up to day 13 (*t*_rec_) in water-limited (WL) plants. Figure 1 illustrates the experimental plan. During the experiment, meteorological data were recorded by LAMMA, the Laboratory of Monitoring and Environmental Modelling for Sustainable Development, Florence (http://www.lamma.rete.toscana.it; the weather station was located at the experimental site).

### Soil–plant water relationships

Substrate volumetric water content was measured daily in WW and WL plants by ‘HydroSense’ probes (Campbell Scientific Inc., Logan, UT, USA), which were based on time-domain reflectometry. The relative extractable water in the substrate (REW) was calculated as follows:

$$\text{REW}=\left({\text{SWC}}_{\text{a}}–{\text{SWC}}_{\text{wp}}\right)/\left({\text{SWC}}_{\text{fc}}–{\text{SWC}}_{\text{wp}}\right)$$ (1)

Here SWC_a_ is the water content of the actual substrate in the root zone; SWC_wp_ is the water content of the substrate at the wilting point (9% w/w); and SWC_fc_ is the water content of the substrate at field capacity (30% w/w).

### Leaf analyses

Leaf water potential at pre-dawn and midday (Ψw_pd_ and Ψw_md_, MPa) was determined using two fully expanded leaves from each plant (the leaf plastochron index was between 5 and 7) and a pressure chamber (PMS Instruments Co., Albany, OR, USA). Leaf water content (LWC) was determined using three fully expanded leaves that were excised before dawn (LWC_pd_) and at midday (LWC_md_). Fresh leaves were excised and weighed (FW) and then dried at 80 °C for 48 h, after which dry weight (DW) was determined.

We used the following equation to calculate LWC:

LWC=100×(FW–DW)/DW (2)

The difference between pre-dawn and midday LWC (ΔLWC_pd-md_) was also calculated.

Leaf stomatal conductance (*g*_s_, mol m^–2^ s^–1^), transpiration rate (*E*, mmol H_2_O m^–2^ s^–1^), and net CO_2_ assimilation rate (*A*_max_, µmol CO_2_ m^–2^ s^–1^) were measured using intact, fully expanded leaves (leaf plastochron index was either 6 or 7) between 13.00 h and 14.00 h with a portable open photosynthesis system (ADC-LCA3, Analytical Development, Hoddesdon, UK). This system operated at a flow rate of 5.7 ml s^–1^ and an ambient CO_2_ pressure of 33 ± 1 Pa. Photosynthetic flux density was >1200 µmol m^–2^ s^–1^. Instantaneous water-use efficiency (WUE*i*, mmol CO_2_ mol H_2_O^–1^) was determined as instantaneous leaf transpiration efficiency, *A*_max_/*E*. Specific leaf area (SLA, m^2^ kg^–1^) was calculated at *t*_max_.

### Stem analyses

Variations in stem radius were monitored with automatic point dendrometers that were constructed for use with small stems. The dendrometers consisted of a linear variable transducer (RS Pro LM10 Series, Rs Component s.r.l., Italy) used to measure linear displacement of a stainless-steel sensing rod (effective travel 10 ± 0.5 mm, linear thermal expansion coefficient 2.5*×*10^–6^ K^–1^), which was pressed against the bark. The transducer was mounted on a polytetrafluoroethylene frame attached to the stem by three titanium rods at 10–15 cm above the ground. The frame was anchored to a polytetrafluoroethylene holder attached on two sides of the pot. As the stem expanded or contracted, the rod transmitted the signal to the transducer. The sensor output, V_1_/V_x_ ratio, was converted into a numerical value (length of sensor in millimetres) using a linear calibration regression equation (Loggernet software, Campbell Scientific Inc.). A total of 16 plants (four plants for each genotype and treatment) were monitored with dendrometers, installed at a quarter of the height of the stem from the collar. Aluminium foil was used to shield all the dendrometers from direct sunlight and weather damage. Raw data were recorded every 15 min, and averages were calculated hourly.

Growth-induced irreversible stem expansion (GRO) and tree water deficit-induced reversible stem shrinkage (TWD) were determined following the procedure reported by Zweifel *et al.* (2016); it was assumed that growth could take place only in the absence of stem radius shrinkage (ZG concept). Moreover, TWD (µm) was defined as the difference between the previous maximum stem radius (SR_max_) and the current stem radius (SR_t_) when SR_t_<SR_max_. Furthermore, GRO (µm) was calculated as the difference between SR_t_ and the highest previous value of SR_max_ when SR_t_>SR_max_.

The rates of stem growth over time (ΔSR/Δ_t_, µm h^–1^) were calculated as follows:

$$\Delta \text{SR}/\Delta_{\text{t}}=\left(\text{GRO}–\text{TWD}\right)/\Delta_{\text{t}}$$ (3)

Instantaneous stem water deficit, ΔW (µm) was extracted by de-trending the original growth data collected from the dendrometers with the help of piecewise linear regression (see Supplementary Fig. S1 at *JXB* online).

At each sampling time, stem portions (50 mm) were excised 20 cm from the collar. The pith was discarded immediately after separating the bark from the xylem. Samples were weighed within 5 min after harvest to determine fresh mass (M_f_) and then fresh volume (V_f_) by water displacement (Haworth *et al.*, 2017). Dry mass (M_d_) was determined after 96 h at 103 °C. Relative water content (RWC, %) was determined for the bark and the xylem using the following formula:

$$\text{RWC}=\left({\text{M}}_{\text{f}}–{\text{M}}_{\text{d}}\right)/\left({\text{V}}_{\text{f}}–{\text{V}}_{\text{d}}\right)\times \text{1}00$$ (4)

Here, dry volume (V_d_) was estimated by dividing M_d_ by 1.53; the density value was assumed for dry cell wall material (Pallara *et al.*, 2012).

Wood moisture content (α) was determined according to Berry and Roderick (2005) at each sampling. Basic wood density was defined as the ratio between the dry mass and the saturated volume (Rosner, 2017). Briefly, stem discs of 8–10 mm were collected, and two prismatic subsamples of xylem were taken by removing the pith and bark. Samples were weighed immediately, and dry mass was calculated after 96 h at 103 °C. To record the saturated volume, fresh woody samples were immersed in distilled and degassed water under a partial vacuum condition (10 kPa) for 72 h.

Roderick and Berry (2001) proposed the following formula to calculate the fibre saturation point (α_f_):

$${\text{α}}_{f}=\frac{0.22}{\sqrt{\frac{\mathrm{basic}\;\mathrm{density}}{\rho_{\text{w}}}}}$$ (5)

Here, ρ_*w*_ is the density of liquid water (kg m^–3^).

The difference α–α_f_ is the amount of free water available to support the hydraulic network.

### Cambial region and mature xylem sampling

The stems were divided into logs of 10–15 cm; these were immediately frozen in liquid nitrogen and then subjected to freeze-drying under vacuum. To obtain samples from the cambial region, the bark was detached from the logs, and differentiating phloem and xylem were gently scraped from the inner side of the bark and the outermost side of the xylem. The xylem was converted to a fine powder using an Ultra Centrifugal Mill ZM 200 (Retsch, Haan, Germany). The complete procedure has been reported in Giovannelli *et al.* (2011).

### Soluble carbohydrate and starch quantification

According to a procedure reported by Giovannelli *et al.* (2011), soluble carbohydrate and starch analyses were performed on the cambial region and the xylem; the extraction was modified using water (Milli Q grade, pH 7) as the extraction solvent. Briefly, the sugar content was determined by HPLC equipped with a SHODEX SUGAR Series SC-1011 8 × 300 mm column (Showa Denko, Germany), which was preceded by a pre-column Sugar-Pak II Guard-Pak Insert (Waters). Water (Milli Q grade) was used for the mobile phase, with a flow rate of 0.5 ml min^–1^. Soluble sugars were identified with a refractive index detector (LC-30 RI, Perkin Elmer); carbohydrate standards were used to corroborate the identified sugars (Sigma-Aldrich, St. Louis, MO, USA). Finally, sorbitol was used to normalize sugar amounts (Harris, 1997). After extracting the soluble sugars, the starch in the remaining powder was analysed.

The contribution to the osmotic potential of sucrose, glucose, and fructose was determined using the following formula (Gucci *et al.*, 1997):

$$\psi \pi_{\text{sat}}\left(\text{sucrose}/\text{fructose}/\text{glucose}\right)=\text{RT}\times \left(\text{RDW}\right)\times \text{C}$$ (6)

Here, Ψπ_sat_ indicates the contribution (MPa) of individual sugars to Ψπ; RDW is the relative dry weight at saturation (kg m^–3^); C is the molar concentration of solutes (mol kg^-1^); and the RT value at 25 °C is –0.002479 m^3^ MPa^–1^ mol^–1^.

### Determination of osmotically active molecules

At each sampling, osmotically active solutes were determined following the procedure described in a study conducted by Arend and Fromm (2007). Dried powders (4 mg) of the cambial region and the xylem were suspended in 250 μl of distilled water. Samples were vortexed and sonicated for 10 min, then centrifuged at 10 000 *g* for 5 min, and supernatants were analysed using a freezing point osmometer (Osmomat 030 Gonotec, Germany). The results were expressed in mOsm g^–1^ DW.

### Histological observations

At each sampling, stem discs of 10–20 mm thickness were cut 30 cm from the collar. They were then placed in ethanol (96%) and stored at 4 °C. Next, disc sections were fixed with ice on a Peltier plate. Finally, these sections were cut into transverse or radial sections of 8–12 μm thickness with a microtome. The sections were stained with Lugol’s solution (Sigma-Aldrich) and observed with a Nikon Eclipse E800 light microscope (Melville, NY, USA).

### Primer design

We evaluated mRNA transcription of the following genes: B-type cyclin *CycB1.3-4* and *CycB2.1*; amylase *Amy1.1-2* and *Bam5*; starch synthase *SS2* and *SS4*; sucrose synthase *Sus2* and *Sus3*; and sugar transporter *Suc2.1*, *SUT2.a-b*, and *SUT4*. Primers for the housekeeping genes *Act-2* and *Ef-1* were obtained according to Brunner *et al.* (2004) and Pallara *et al.* (2012), respectively. Primers for *CycB1.3-4* and *CycB2.1* genes were obtained from Emiliani *et al.* (2016). New primers were designed for the other genes. To enable primer design, nucleotide sequences of *P. trichocarpa* genes were retrieved from the PopGenIE database (www.popgenie.org;Sjödin *et al.*, 2009) using the BLAST search tool. Sequences of homologous genes of *A. thaliana* were retrieved from the TAIR database (www.arabidopsis.org;Huala *et al.*, 2001). Sequences showing significant hits were aligned with Muscle (Edgar, 2004) and trimmed to eliminate poorly aligned regions. A Neighbor–Joining tree (1000 bootstrap) was built using the MEGA6 program (Tamura *et al.*, 2011). To construct a dendrogram for sucrose synthase genes, sequence and isoform nomenclatures were reported in a study conducted by Zhang *et al.* (2011). For the identification of sucrose transporter genes, the same nomenclature as adopted by Secchi and Zwieniecki (2011) was used. The principal features of the primers used are reported in Supplementary Table S1.

### RNA isolation and RT–PCR analysis

According to a procedure described by Chang *et al.* (1993), total RNA was extracted from the cambial region powder in three biological replicates for each treatment and time. The extraction methodology was adjusted by replacing spermidine with β-mercaptoethanol in the extraction buffer. The quality of RNA was verified by performing agarose gel electrophoresis. Then, RNA was quantified using a NanoDrop 2000 spectrophotometer (Thermo Fisher Scientific, Waltham, MA, USA). To eliminate DNA contamination, 3 μg of total RNA were treated with DNase using the DNase I Amplification Grade kit (Invitrogen, Thermo Fisher Scientific) according to the manufacturer’s instructions. After treatment with DNase, RNA was precipitated with LiCl and re-suspended in 50 μl of diethylpyrocarbonate- (DEPC) treated water. Using the reverse transcription–PCR method, RNA was retro-transcribed into first-strand cDNA with SuperScript VILO™ MasterMix (Invitrogen, Thermo Fisher Scientific) according to the manufacturer’s directions. The reaction was performed with random primers in a 25 μl final volume. To determine the mRNA accumulation of target genes, cDNA was used in RT–PCR (details of the primer design are given in Supplementary Table S1). Using the ΔΔCq method (Pfaffl, 2001), the results were compared with the reference genes, *Act-2* and *Ef-1*. Target and reference gene-specific amplifications were calculated based on quantification cycles (Cqs) of all cDNA samples. For each gene and each poplar genotype, a five-point dilution set of two standard samples was amplified in duplicate reactions, and reaction efficiency was verified. Then, RT–PCRs were carried out in duplicate reactions for each sample; outcomes were discarded if the difference in the Cq numbers was >0.5 for the replicates. For each gene, a no-template control was amplified in each run. For all primers, RT–PCRs were conducted with an annealing temperature of 60 °C using the iTaq™ Universal SYBR^®^ Green Supermix kit (Bio-Rad, California, USA). In a final volume of 10 μl, the reaction was performed with 10 μM of each primer and 1 μl of a 1:5 dilution of template cDNA. The RT–PCR protocol was as follows: 95 °C for 5 s and 60 °C for 30 s; a total of 40 amplification cycles were performed. At the end of the amplification cycles, the melting curve was calculated for each amplification.

### Statistical analyses

All analyses were performed on four biological replicates (*n*=4). Then, RT–PCR analysis was performed on three biological replicates (*n*=3). Data were checked for normal distribution (D’Agostino–Pearson’s *K*^2^ test). The effects of the water regime and genotype were assessed with two-way ANOVA. Post-hoc analysis was conducted using Fisher’s LSD test. Furthermore, *t*-test analysis was performed at sampling time *t*_0_ (*n*=3). Data that did not have a normal distribution and percentages were transformed before ANOVA. Correlation was evaluated by performing Pearson’s test. All analyses were executed using NCSS Data Analysis software. All graphs were plotted using Prism 5 software (GraphPad, La Jolla, CA, USA).

## Results

### Physiological and biochemical comparisons of poplar genotypes under well-watered conditions

**Fig. 2.**
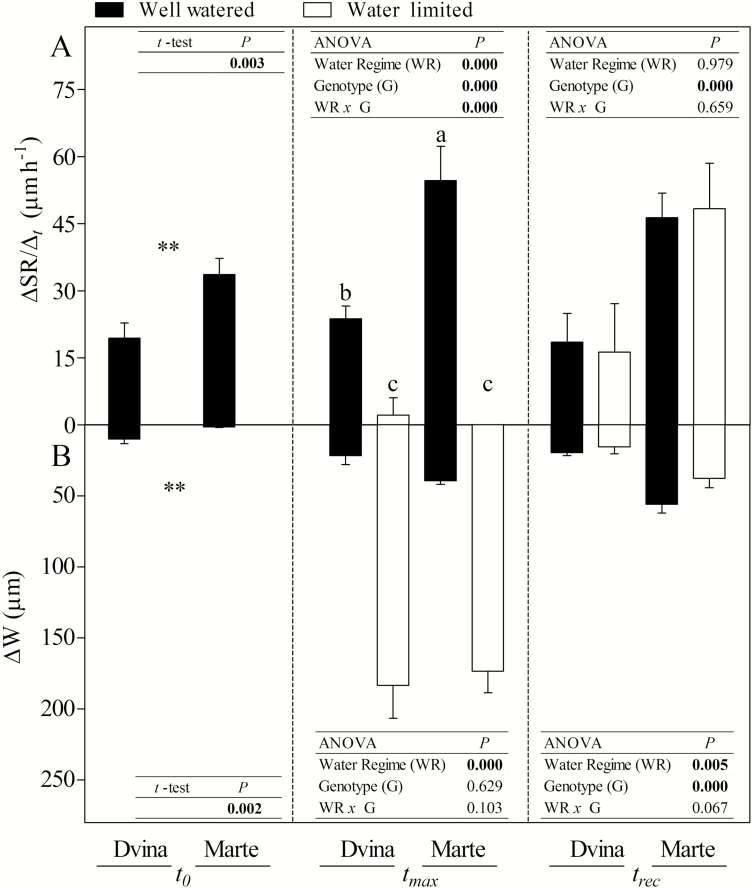
Stem growth rate (ΔSR/Δ_t_) (A) and instantaneous stem water deficit (ΔW) (B) of *P. deltoides* clone ‘Dvina’ and *P. alba* clone ‘Marte’ subjected to 8 d of water deficit. Data were compared between genotypes and treatments at *t*_0_ (0 d), after 8 d of withholding water (*t*_max_), and 13 d after resumption of irrigation (*t*_rec_). The bars represent the mean of four biological replicates (±SD). Data were analysed with *t*-test (*t*_0_) or two-way ANOVA (*t*_max_ and *t*_rec_). Significant differences are shown in bold. The means were compared using Fisher’s LSD test. Different letters indicate significant differences (*P*<0.05).

**Fig. 3.**
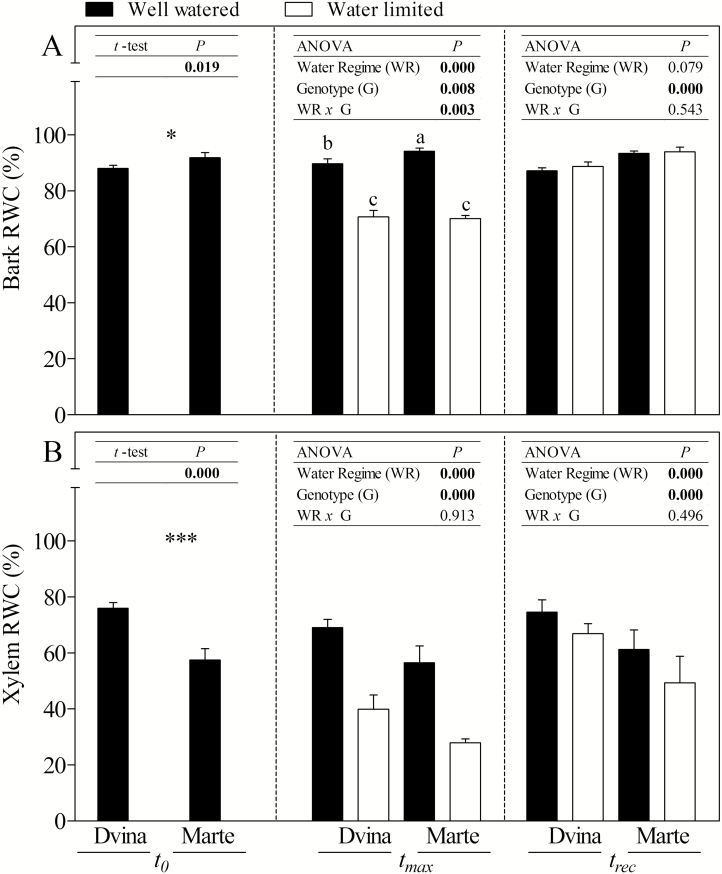
Bark (A) and xylem (B) relative water contents of *P. deltoides* clone ‘Dvina’ and *P. alba* clone ‘Marte’ at *t*_0_ (0 d), after 8 d of withholding water (*t*_max_), and 13 d after resumption of irrigation (*t*_rec_). The bars represent the mean of four biological replicates (±SD). For the statistical analyses, the percentages were subjected to arcsine transformation (arcsine √*x*). Data were analysed with *t*-test (*t*_0_) or two-way ANOVA (*t*_max_ and *t*_rec_). Significant differences are shown in bold. The means were compared using Fisher’s LSD test. Different letters indicate significant differences (*P*<0.05).

Under WW conditions, the two genotypes ‘Dvina’ and ‘Marte’ displayed different morpho-physiological and biochemical traits that highlighted contrasting growth performances and water–carbon balance strategies. The genotype ‘Marte’ showed a higher rate of growth than the genotype ‘Dvina’, both in stem growth rate (ΔSR/Δ_t_, Fig. 2A) and in stem elongation (1.6 mm d^–1^ versus 1.0 mm d^–1^, data not shown). Compared with ‘Dvina’, ‘Marte’ had a higher RWC in the bark (Fig. 3A, 88% versus 92%, respectively) and a lower instantaneous stem water deficit (ΔW, Fig. 2B). In contrast, RWC (Fig. 3B) and the basic density (366.6 kg m^–3^ in ‘Divina’ versus 306.5 kg m^–3^ in ‘Marte’, *P*=0.003) of the xylem were significantly higher in ‘Dvina’ than in ‘Marte’. However, the amount of free water available to support the hydraulic network (α–α_f_) did not differ significantly in the two genotypes (Table 2).

Under WW conditions, the leaves of ‘Marte’ displayed a higher specific leaf area and ΔLWC_pd-md_ than ‘Dvina’, while no significant changes were observed in leaf ΔΨw_pd-md_ and WUE*i* between the genotypes (Table 2). Genotypes displayed different carbohydrate concentrations within the cambial region and xylem (Table 1). ‘Dvina’ had significantly more starch than ‘Marte’ within the xylem, and this accumulation was even higher in the cambial region. The highest concentration of starch was corroborated by histological observation with Lugol’s solution (Supplementary Fig. S2A–C). In contrast, ‘Marte’ contained a higher concentration of soluble sugars, mostly glucose, in the cambial region. Finally, ‘Marte’ had a higher total Ψπ_sat_ than ‘Dvina’ (Table 1; Supplementary Table S2).

**Table 1.** Sucrose and starch content, sucrose Ψπ_sat_, and sucrose:starch ratio in the cambial region and xylem of *P. deltoides* ‘Dvina’ and *P. alba* ‘Marte’ at *t*_0_ (0 d), after 8 d of withholding water (*t*_max_) and 13 d after resumption of irrigation (*t*_rec_)

Parameter	Genotype (G)						
	‘Dvina’		‘Marte’				
	Water regime (WR)						
	WW		WW				
*t*_0_	Cambial region				*t*-test		
Starch (mg g^–1^ DW)	109.3 ± 20.4		11.6 ± 2.7		***		
Sucrose (mg g^–1^ DW)	84.8 ± 14.3		93.7 ± 33.3		ns		
Ψπ_sat_ sucrose (MPa)	–0.23 ± 0.02		–0.21 ± 0.05		ns		
Sucrose:starch	0.9 ± 0.2		6.2 ± 2.5		ns		
	*Xylem*						
Starch (mg g^–1^ DW)	10.0 ± 1.4		3.6 ± 2.3		**		
Sucrose (mg g^–1^ DW)	21.9 ± 6.2		21.6 ± 3.1		ns		
Ψπ_sat_ sucrose (MPa)	–0.08 ± 0.02		–0.06 ± 0.01		ns		
Sucrose:starch	2.2 ± 0.6		7.4 ± 3.7		ns		
	‘Dvina’		‘Marte’		ANOVA		
	WW	WL	WW	WL	WR	G	WR*×*G
*t*_max_	Cambial region						
Starch (mg g^–1^ DW)	79.9 ± 46.0	51.2 ± 12.9	18.2 ± 7.6	34.9 ± 18.1	ns	**	ns
Sucrose (mg g^–1^ DW)	50.0 ± 13.2 b	60.3 ± 12.4 ab	73.6 ± 12.0 a	55.2 ± 7.7 b	ns	ns	*
Ψπ_sat_ sucrose (MPa)	–0.15 ± 0.04 b	–0.18 ± 0.04 ab	–0.21 ± 0.03 a	–0.16 ± 0.02 b	ns	ns	*
Sucrose:starch	0.9 ± 0.6 b	1.2 ± 0.3 b	4.4 ± 1.1 a	2.0 ± 1.1 b	*	***	**
	Xylem						
Starch (mg g^–1^ DW)	9.7 ± 2.5	8.2 ± 1.9	7.9 ± 1.8	3.9 ± 1.7	*	*	ns
Sucrose (mg g^–1^ DW)	20.5 ± 3.9	22.8 ± 2.8	22.1 ± 4.7	29.8 ± 1.7	*	*	ns
Ψπ_sat_ sucrose (MPa)	–0.08 ± 0.01	–0.08 ± 0.01	–0.06 ± 0.01	–0.09 ± 0.00	*	ns	ns
Sucrose:starch	2.0 ± 0.7 b	2.9 ± 0.8 b	2.9 ± 0.9 b	8.6 ± 3.3 a	**	**	*
*t*_rec_	Cambial region						
Starch (mg g^–1^ DW)	95.84 ± 25.0	70.4 ± 26.2	13.8 ± 13.2	39.0 ± 18.7	ns	**	ns
Sucrose (mg g^–1^ DW)	61.6 ± 14.7 c	80.3 ± 12.5 b	100.5 ± 15.7 a	76.9 ± 9.8 bc	ns	*	*
Ψπ_sat_ sucrose (MPa)	–0.18 ± 0.04 b	–0.23 ± 0.04 ab	–0.28 ± 0.04 a	–0.22 ± 0.03 ab	ns	ns	*
Sucrose:starch	0.7 ± 0.3 b	1.3 ± 0.6 b	11.5 ± 6.7 a	2.2 ± 0.7 b	*	**	*
	Xylem						
Starch (mg g^–1^ DW)	19.9 ± 2.7	24.1 ± 1.8	12.1 ± 1.4	14.4 ± 4.2	*	***	ns
Sucrose (mg g^–1^ DW)	23.8 ± 9.0	14.6 ± 2.7	19.7 ± 2.6	19.0 ± 2.7	ns	ns	ns
Ψπ_sat_ sucrose (MPa)	–0.09 ± 0.03	–0.05 ± 0.01	–0.06 ± 0.01	–0.05 ± 0.01	ns	ns	ns
Sucrose:starch	1.2 ± 0.5	0.6 ± 0.5	1.7 ± 0.4	1.4 ± 0.4	*	**	ns

The values represent the mean of four biological replicates ±SD. Data were analysed with *t*-test (*t*_0_) or two-way ANOVA (*t*_max_ and *t*_rec_). The means were compared using Fisher’s LSD test. Different letters indicate significant differences (*P*<0.05). WW, well watered; WL, water limited; ns, not significant; *P<0.05; **P<0.01; ***P<0.001.

### Physiological and biochemical comparisons of poplar genotypes under water-limited conditions and following rehydration

**Fig. 4.**
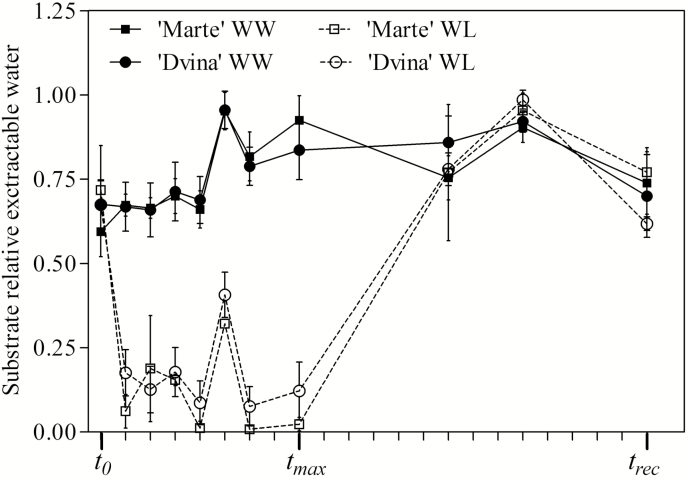
Relative extractable water (REW) in the substrate of *P. deltoides* clone ‘Dvina’ and *P. alba* clone ‘Marte’ during the experiment. The values were referred at *t*_0_ (0 d), after 8 d of withholding water (*t*_max_), and 13 d after resumption of irrigation (*t*_rec_). The values represent the mean of four biological replicates (±SD).

The intensity of a water deficit was determined from the relative extractable water of the substrate (Fig. 4). The results indicate that both genotypes were subjected to the same water deficit intensity and duration, which was in the relative extractable water range of 0.05 and 0.40 (genotype effect, 0.060≤*P*≤0.617). Despite the similar water deficit, following 8 d of suspension of irrigation (*t*_max_), several traits were affected to different extents, depending on the genotype. Stem elongation (data not shown) was negatively affected in ‘Dvina’ (*P*=0.004), but not in ‘Marte’ (*P*=0.220). In contrast, ΔSR/Δ_t_ (Fig. 2A) was zero in ‘Marte’ WL plants, while a reduction of 91% was detected in ‘Dvina’ WL versus WW plants.

On the whole, ‘Marte’ showed more variations in physiological and biochemical parameters. In ‘Marte’, a significant reduction of ΔLWC_pd-md_ (–37%) was recorded in WL plants compared with WW plants; however, no significant differences were observed in ‘Dvina’ plants. In addition, ‘Dvina’ WL plants maintained a significantly higher leaf ΔΨw_pd-md_ than ‘Marte’ (0.5 MPa versus 0.1 MPa, respectively). Both genotypes showed a decrease in RWCs of the bark and xylem (Fig. 3), an increase in instantaneous stem water deficit (ΔW, Fig. 2B), and a decrease in α–α_f_ (Table 2).

**Table 2.** Water potential differences between pre-dawn and midday (ΔΨw_pd-md_, MPa), instantaneous water-use efficiency (WUE*i*, mmol CO_2_ mol^–1^ H_2_O), leaf water content difference between pre-dawn and midday (ΔLWC_pd-md,_ %), specific leaf area (SLA, m^2^ kg^–1^), and the amount of free water available to support the hydraulic network (α–α_f_) of *P. deltoides* ‘Dvina’ and *P. alba* ‘Marte’ at *t*_max_

Parameter	Genotype (G)				ANOVA		
	‘Dvina’		‘Marte’				
	Water regime (WR)						
	WW	WL	WW	WL	WR	G	WR*×*G
ΔΨw_pd-md_	1.2 ± 0.1 a	0.5 ± 0.2 b	1.2 ± 0.1 a	0.1 ± 0.1 c	***	*	*
WUE*i*	1.2 ± 0.1	0.2 ± 0.0	1.4 ± 0.1	0.4 ± 0.3	***	ns	ns
ΔLWC_pd-md_	5.1 ± 0.3 b	6.6 ± 4.0 b	37.4 ± 3.9 a	0.0 ± 6.5 b	***	**	***
SLA	9.6 ± 1.0	10.6 ± 1.8	12.7 ± 1.0	12.5 ± 1.1	ns	***	ns
α–α_f_	1.03 ± 0.10 a	0.44 ± 0.07 b	1.05 ± 0.06 a	0.30 ± 0.01 c	***	ns	*

The values represent the mean of four biological replicates ± SD. Data were analysed with two-way ANOVA. The means were compared using Fisher’s LSD test. Different letters indicate significant differences (*P*<0.05). WW, well watered; WL, water limited; ns, not significant; *P<0.05; **P<0.01; ***P<0.001.

Water deficits induced contrasting effects on genotype in regard to soluble sugar and starch contents within the xylem and cambial region (Table 1; Supplementary Table S2). Starch content decreased, while sucrose content increased significantly in the xylem of ‘Marte’ WL plants. These modifications were accompanied by a significant increase in sucrose Ψπ_sat_ and a higher sucrose:starch ratio (*P*<0.05; *t*-test between WW and WL plants). In contrast, the sucrose content decreased significantly in the cambial region of ‘Marte’ (*P*<0.05; *t*-test between WW and WL plants). Compared with WW plants, no variations were observed in the soluble sugars and starch of ‘Dvina’ WL plants.

Thirteen days from the resumption of irrigation (*t*_rec_), both genotypes demonstrated a complete restoration of primary and secondary growth, ΔSR/Δ_t_, ΔW, bark RWC (Figs 2, 3A), ΔLWC_pd-md_, leaf ΔΨw, and WUE*i* (data not shown). The only exception was the RWC of the xylem (Fig. 3B), which was lower in WL plants than in WW plants. Water content was incompletely restored in this compartment (*P*<0.05; *t*-test between WW and WL plants for both genotypes). With regard to carbohydrates (Table 1; Supplementary Table S2), sucrose increased in the cambial region of ‘Dvina’ (+15% on average) but decreased in ‘Marte’. During the experiment, the concentration of starch increased in the parenchymal cells of xylem in both genotypes and under both treatments. This increase was corroborated by histological observation (Supplementary Fig. S2B–D).

During the experiment, soluble sugars and osmotically active molecules showed a significant correlation in the xylem of ‘Marte’ (*R*=0.837, *P*<0.001; Supplementary Fig. S3), which was not observed in ‘Dvina’.

### Gene transcription

**Fig. 5.**
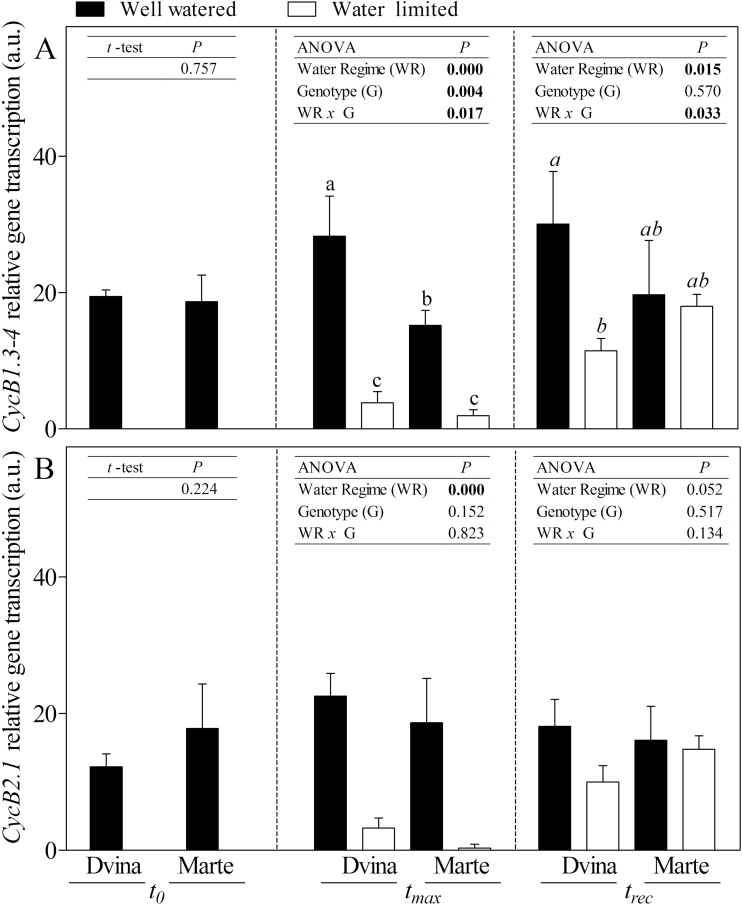
mRNA accumulations of *CycB1.3-4* (A) and *CycB2.1* (B) genes in the cambial region of *P. deltoides* ‘Dvina’ and *P. alba* ‘Marte’ at *t*_0_ (0 d), after 8 d of withholding water (*t*_max_), and 13 d after resumption of irrigation (*t*_rec_). The bars represent the mean of three biological replicates (±SD). Data were analysed with *t*-test (*t*_0_) or two-way ANOVA (*t*_max_ and *t*_rec_). Significant differences are shown in bold. The means were compared using Fisher’s LSD test. Different letters indicate significant differences (*P*<0.05).

The mRNA accumulations of target genes showed genotype- and treatment-dependent responses. Cyclin B genes, specifically *CycB1.3-4* and *CycB2.1* isoforms (Fig. 5A and B, respectively), showed a very strong decrease of transcription at *t*_max_ in WL plants. Moreover, the *CycB1.3-4* gene basal level of transcription was not restored at *t*_rec_ in ‘Dvina’ WL plants (Fig. 5B). Two-way ANOVA indicated that B1 type cyclin was more influenced by genotype and water regime than B2 type cyclin.

**Fig. 6.**
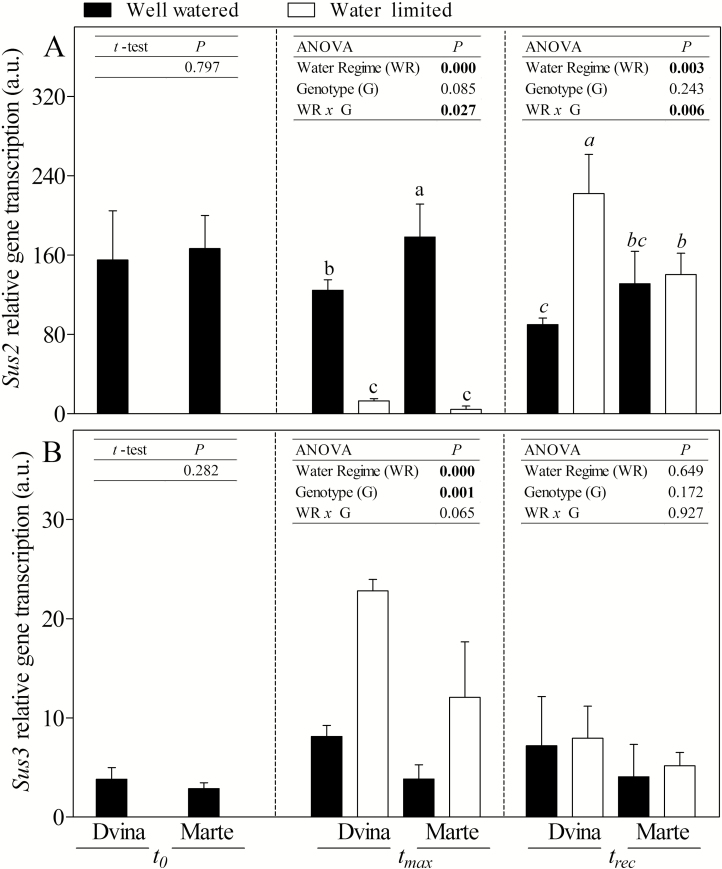
mRNA accumulations of *Sus2* (A) and *Sus3* (B) genes in the cambial region of *P. deltoides* ‘Dvina’ and *P. alba* ‘Marte’ at *t*_0_ (0 d), after 8 d of withholding water (*t*_max_), and 13 d after resumption of irrigation (*t*_rec_). The bars represent the mean of three biological replicates ±SD. Data were analysed with *t*-test (*t*_0_) or two-way ANOVA (*t*_max_ and *t*_rec_). Significant differences are shown in bold. The means were compared using Fisher’s LSD test. Different letters indicate significant differences (*P*<0.05).

**Fig. 7.**
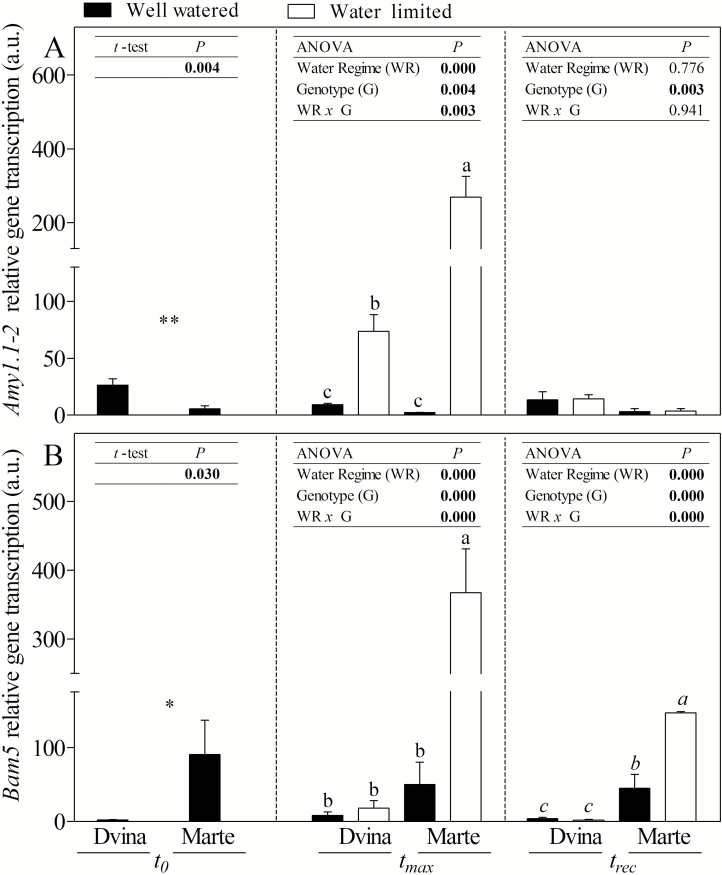
Accumulation of mRNAs of *Amy1.1-2* (A) and *Bam5* (B) genes in the cambial region of *P. deltoides* ‘Dvina’ and *P. alba* ‘Marte’ at *t*_0_ (0 d), after 8 d of withholding water (*t*_max_), and 13 d after resumption of irrigation (*t*_rec_). The bars represent the mean of three biological replicates ±SD. Data were analysed with *t*-test (*t*_0_) or two-way ANOVA (*t*_max_ and *t*_rec_). Significant differences are shown in bold. The means were compared using Fisher’s LSD test. Different letters indicate significant differences (*P*<0.05).

Except for *Sus2* and *Bam5* genes, sucrose/starch pathway-related genes showed similar trends in both genotypes. In general, a higher response was recorded in ‘Marte’. *Sus* isoforms (Figure 6A, B) showed an opposite trend during water deficit. At *t*_max_ in WL plants, *Sus2* was strongly down-regulated while *Sus3* was up-regulated. An up-regulation in ‘Dvina’ WL plants was observed only for the *Sus2* gene at *t*_rec_. The *Amy1.1-2* gene was more transcribed in both WL genotypes, mostly in ‘Marte’ (Fig. 7A). In contrast, *Bam5* was strongly transcribed in ‘Marte’ WL plants, but not in ‘Dvina’ (Fig. 7B).

**Fig. 8.**
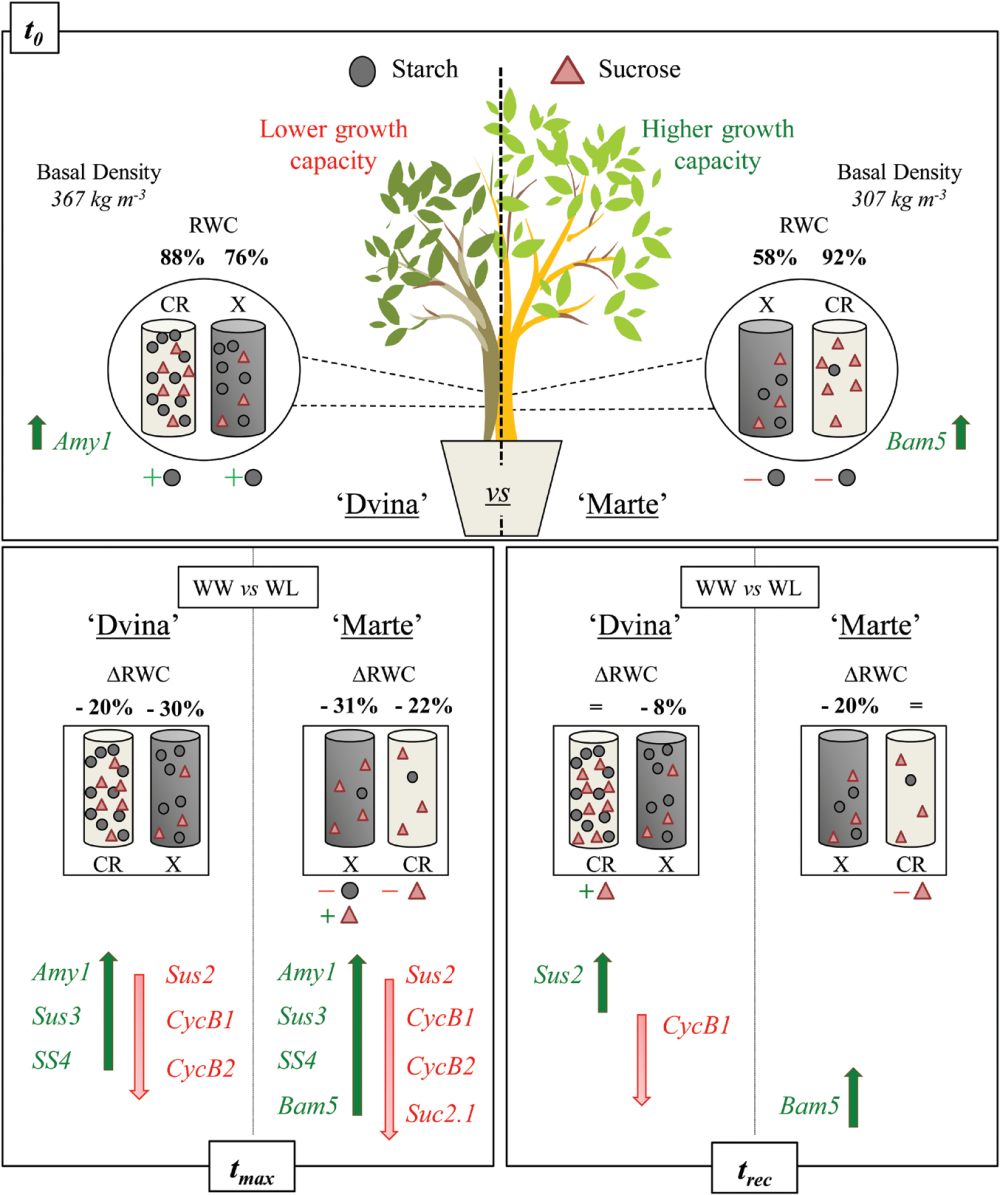
Schematic overview of experimental results on *P. deltoides* ‘Dvina’ and *P. alba* ‘Marte’ at *t*_0_ (0 d), after 8 d of withholding water (*t*_max_), and 13 d after resumption of irrigation (*t*_rec_). CR, cambial region; X, xylem; WW, well-watered; WL, water limited.

The other analysed genes showed a less marked response during water deficit. Moreover, *SS2* and *SS4* (Supplementary Fig. S4A and B, respectively) showed a weakly higher transcription at *t*_max_ in WL plants, especially *SS4*. In a few cases, sucrose transporter genes *SUT2a-b*, *Suc2.1*, and *SUT4* (Supplementary Fig. S5A–C, respectively) showed a slightly lower transcription in WL plants, especially *Suc.2.1* in ‘Marte’. A schematic overview of experimental results is reported in Fig. 8.

## Discussion

Two genotypes, *P. alba* ‘Marte’ and *P. deltoides* ‘Dvina’, demonstrated different responses to water deficits. The results indicate that ‘Marte’ was more anisohydric than ‘Dvina’. A water-consuming strategy, which is typical of anisohydric plants under drought conditions, was related to significant differences between pre-dawn and midday leaf water potentials and relative water contents (Attia *et al.*, 2015). In ‘Marte’, this response aligned with the greatest plasticity in the sucrose/starch pathway. These divergent strategies highlighted the differential involvement of non-structural carbohydrates in the drought tolerance of poplar. This could explain the rapid growth recovery of ‘Marte’ following water deficit, which was related to variations in sugar metabolism.

Soluble and stored carbohydrates did not represent a limiting factor for growth during water deficit. In ‘Marte’, the increase of sucrose in the xylem and the decrease of starch in the xylem and sucrose in the cambial region were clear indications that non-structural carbohydrates were involved in osmotic adjustments. These changes mirrored those recorded for sucrose and starch in the parenchymal cells during an embolism event, as reported by Secchi and Zwieniecki (2011) in *P. trichocarpa* and by Salleo *et al.* (2009) in *Laurus nobilis*. In fact, there was a correlation between soluble sugars and osmotically active compounds in the xylem of ‘Marte’. This highlights the importance of carbohydrates as active osmotic molecules, which were involved in eliciting a water deficit response in this genotype. In ‘Marte’, a large amount of soluble carbohydrates was present within the stem; however, these sugars were not stored as starch. This could represent an important amount of carbon for supporting cambial cell division during recovery (Oribe *et al.*, 2003). Water contained within the phloem structures could be moved into the xylem vessels to sustain the transpiration stream and alleviate the risk of cavitation (Pfautsch *et al.*, 2015). In WW conditions, the higher RWC of the bark of ‘Marte’ might represent a reserve of water, which was more abundant than in ‘Dvina’; this could be used during periods of water deficit. Within the xylem–phloem compartment, the high amount of water and carbohydrate content was associated with the maintenance of stem elongation in ‘Marte’ during water deficit conditions. Thus, in this genotype, primary meristems (apex) had a higher C-sink strength than the cambial region under water deficit. However, the bark RWC decreased to 70% in both genotypes at *t*_max_. This indicates that this level of water could represent a threshold value for the functionality of phloem in poplar species. In ‘Marte’, osmotic adjustments were activated by decreasing the water reserve within the phloem; this was achieved by fluctuating stored and non-stored non-structural carbon compounds in the xylem and the cambial region. Mencuccini *et al.* (2013) proposed that the osmotic potential of the phloem can influence the radial transfer of water and variations in bark thickness. The response of ‘Marte’ confirmed a strong decrease in stem growth rate and an increase in instantaneous water deficit, which was mirrored by a decrease in sucrose content and Ψπ_sat_ in the cambial region.

In the cambial region of ‘Marte’, a high amount of soluble carbohydrates was associated with a higher transcription of the gene *Bam5*. This gene was involved in the degradation of starch; thus, a very rapid carbon turnover might consequently be achieved. The gene *Bam5* was overexpressed in several different starchless mutants of *A. thaliana* (Monroe *et al.*, 2014), indicating that different starch contents found in our contrasting genotypes could be strictly related to different transcription levels of this gene. In ‘Marte’, *Bam5* gene transcription was increased strongly during water deficit and recovery. This highlighted the probable pivotal function of this gene in the differential activation of osmotic adjustments fluctuating stored and non-stored non-structural carbohydrates. However, in both genotypes, starch was accumulated in the experimental period (from July to August), confirming that storage was not influenced by water deficit but was driven mainly by cambium phenology (Sauter and Van Cleve, 1994; Regier *et al.*, 2010).

A higher transcription of the *Sus2* gene occurred after rehydration in ‘Dvina’. Konishi *et al.* (2004) and Coleman *et al.* (2009) proposed that the *Sus2* isoform was responsible for directing carbon towards cellulose biosynthesis in poplar species. This could imply that C-fluxes were directed mainly towards cellulose production, corroborating the observation previously made by Cocozza *et al.* (2011) in the same genotype under similar conditions. In the long term, the high carbon investment in the cell wall matrix, as recorded in ‘Dvina’, could reflect an increase in wood density. This induced a higher resistance to embolism formation and decreased the risk of cell wall collapse (Jacobsen *et al.*, 2005). However, the increase in wood density was also related to the poor technological proprieties of newly produced wood (Cocozza *et al.*, 2011). The low wood density of ‘Marte’ was related to rapid growth and more anisohydric behaviour of this genotype. The same traits were also observed in a study conducted by Eller *et al.* (2018) on tropical trees. In ‘Marte’, the difference between pre-dawn and midday leaf water potential and water content was higher. This strongly decreased free water, which was available to support the hydraulic network (α–α_f_) in the xylem. The high ‘risk-taking’ behaviour of ‘Marte’ under water deficit was coupled with similar carbohydrate variations, which were also observed in other woody plants during embolism response (Salleo *et al.*, 2009; Secchi and Zwieniecki, 2011; Attia *et al.*, 2015).

B-type cyclins regulated a crucial checkpoint for mitosis (Fowler *et al.*, 1998), which was the primary activity in the cambial region and the starting point of new xylem production (Larson, 1994). Under WL conditions, B-type cyclins were strongly down-regulated while carbohydrate content increased. This supported the theory that cell turgor prevented growth regardless of the carbon supply (Palacio *et al.*, 2014). The assumption of no growth during stem shrinkage, described as the ZG concept by Zweifel *et al.* (2016), was corroborated by synergic analyses of B-type cyclin transcription and stem radius variations. In fact, the results confirmed that when the stem did not grow (ΔSR/Δ_t_*=*0), the transcription levels of *CycB1.3-4* and *CycB2.1* genes were at the detection limit.

In conclusion, several physiological features were associated with different genotype-dependent responses. These features were related to the capacity to reallocate carbohydrates during water deficit. Moreover, the importance of cambial region activity under water deficit was clearly demonstrated and related to xylem traits. ‘Marte’ showed an active response and a rapid recovery following water deficit, which was related to greater plasticity in sugar/starch conversion. In contrast, less anisohydric behaviour was demonstrated by ‘Dvina’ during water deficit. This might be related to the inability to use carbohydrates in the osmotic processes. These carbohydrates were preferentially stored or used for cellulose production.

To elucidate plant responses to water deficits, it is necessary to understand how plants’ water status and leaf gas exchange can be integrated with the transportation of photoassimilates within the phloem and xylem. Moreover, basic information is required about daily carbon balances and the limits of plants to shift energy among competing sinks under water-limited conditions.

## Supplementary Data

Supplementary data are available at *JXB* online.

Table S1. List of primers used in this research.

Table S2. Glucose and fructose contents, and glucose and fructose Ψπ_sat_ in the cambial region and the xylem.

Fig. S1. Calculation of the instantaneous stem water deficit.

Fig. S2. Starch localization using Lugol’s solution.

Fig. S3. Osmotically active molecules in the cambial region and xylem.

Fig. S4. Accumulation of mRNAs *SS2* and *SS4* genes.

Fig. S5. Accumulation of mRNAs of *SUT2 a-b*, *Suc2.1.a-b*, and *SUT4* genes.

Supplementary Material
