# Supplementary material for: Can sugar metabolism in the cambial region explain the water deficit tolerance in poplar?
Source: J Exp Bot. 2018 May 26;69(16):4083–97. doi: 10.1093/jxb/ery195 (PMC6054210; doi:10.1093/jxb/ery195)
Supplement: Supplementary Material [file ery195_suppl_supplementary_materials.pdf]

Electronic Supplementary Material

Can sugar metabolism in the cambial region explain the water deficit tolerance in poplar?

Silvia Traversari[a], Alessandra Francini[a], Maria Laura Traversi[b], Giovanni Emiliani[b], Carlo Sorce[c], Luca Sebastiani[a*], Alessio Giovannelli[a,b]

[a]Institute of Life Sciences, Scuola Superiore Sant'Anna, Piazza Martiri della Libertà, 33, 56127, Pisa, Italy.
[b]Trees and Timber Institute (IVALSA-CNR), Via Madonna del Piano, 10, 50019, Sesto F.no (Florence), Italy.
[c]Department of Biology, University of Pisa, Via Luca Ghini, 13, 56126, Pisa, Italy.

Corresponding Author*:

Luca Sebastiani, luca.sebastiani@santannapisa.it; tel: + 39 050 883070.

Table S1. Primer sequences.

Primer name	Sequence (5'- 3')	Target sequence(s)	Amplicon size (bp)
Sus2 F	ACTGCTGAGCGTGTTCTTCA	Potri.006G136700	221
Sus2 R	TCACTCTCCAAGGCACGAAC		
Sus3 F	CGCTAACCGTAACGTGCTTG	Potri.002G202300	229
Sus3 R	CCAAACTCCAGGTCTCGGAC		
Amy1.1-2 F	TATAACCAGGATGCGCACCG	Potri.014G029400<br>Potri.002G126300	214
Amy1.1-2 R	ACCCGGTGTCATGATTGTCA		
Bam5 F	GAGAACATGGCGGATTTTCTTG	Potri.017G040800	173
Bam5 R	GCTGCCTCTTTGAATTCTGCT		
SS2 F	CTGAAGGTGGTTGGGGTCTG	Potri.017G084100	155
SS2 R	GTGCAGTGTTTCCAGGGAGT		
SS4 F	ATTGATGGTTGGCTGCTCGA	Potri.001G351800	243
SS4 R	GACGTCTCCCAAACCACCAA		
Suc2.1 F	CAACAACAACTGCCCTCCTC	Potri.019G085800	231
Suc2.1 R	GCCCACAGAGCCAAATGAAA		
SUT2a-b F	TGTGAAGCTTGCGGAAATCT	Potri.010G093600<br>Potri.008G148100	131
SUT2a-b R	TCTGATAAATGGCGTGGTTGA		
SUT4 F	ATCCCTCACAAATGGGCCAG	Potri.002G106900	206
SUT4 R	CTATCGCCTAACCACCACCC		

Table S2. Glucose and fructose contents, glucose and fructose $\Psi\pi_{sat}$ in the cambial region and xylem of *P. deltoides* 'Dvina' and *P. alba* 'Marte' at t_0 (0 d), after 8 d of withholding water (t_{max}), and 13 d after resumption of irrigation (t_{rec}). The values represent the mean of four biological replicates ± SD. Data were analysed with *t*-test (t_0) or two-way ANOVA (t_{max} and t_{rec}). The means were compared using Fisher's LSD test. Different letters indicate significant differences ($P < 0.05$). WW, well-watered; WL, water limited; ns, not significant; **P* < 0.05; ***P* < 0.01; ****P* < 0.001.

Parameter	Genotype (G)						
	'Dvina'		'Marte'				
	Water Regime (WR)						
	WW		WW		*t*-test		
t_0	*Cambial Region*						
Glucose (mg g^{-1}DW)	10.1±2.2		21.7±7.7		*		
Fructose (mg g^{-1}DW)	14.3±4.4		17.7±4.6		ns		
$\Psi\pi_{sat}$ glucose (MPa)	-0.06±0.01		-0.12±0.04		*		
$\Psi\pi_{sat}$ fructose (MPa)	-0.08±0.02		-0.10±0.02		ns		
	Xylem						
Glucose (mg g^{-1}DW)	3.6±1.0		5.8±0.8		ns		
Fructose (mg g^{-1}DW)	4.3±0.7		3.3±0.7		ns		
$\Psi\pi_{sat}$ glucose (MPa)	-0.03±0.01		-0.03±0.00		ns		
$\Psi\pi_{sat}$ fructose (MPa)	-0.03±0.00		-0.02±0.00		*		
	'Dvina'		'Marte'		ANOVA		
	WW	WL	WW	WL	WR	G	WR × G
t_{max}	*Cambial Region*						
Glucose (mg g^{-1}DW)	8.1±2.2	11.9±1.1	15.4±6.1	14.6±3.5	ns	*	ns
Fructose (mg g^{-1}DW)	12.1±1.7	14.7±2.0	16.1±8.0	11.0±1.1	ns	ns	ns
$\Psi\pi_{sat}$ glucose (MPa)	-0.04±0.01	-0.07±0.01	-0.08±0.03	-0.08±0.02	ns	*	ns
$\Psi\pi_{sat}$ fructose (MPa)	-0.07±0.01	-0.08±0.01	-0.09±0.04	-0.06±0.01	ns	ns	ns
	Xylem						
Glucose (mg g^{-1}DW)	3.0±1.1	3.9±1.2	3.8±0.6	3.7±1.3	ns	ns	ns
Fructose (mg g^{-1}DW)	3.7±0.9	4.7±0.9	2.8±0.5	5.9±1.6	**	ns	ns
$\Psi\pi_{sat}$ glucose (MPa)	-0.02±0.01	-0.03±0.01	-0.02±0.00	-0.02±0.01	ns	ns	ns
$\Psi\pi_{sat}$ fructose (MPa)	-0.03±0.01	-0.03±0.01	-0.02±0.00	-0.03±0.01	**	ns	ns
	'Dvina'		'Marte'		ANOVA		
	WW	WL	WW	WL	WR	G	WR × G
t_{rec}	*Cambial Region*						
Glucose (mg g^{-1}DW)	5.4±1.1 b	8.9±2.2 b	23.1±4.6 a	18.9±3.0 a	ns	***	*
Fructose (mg g^{-1}DW)	11.6±3.2	16.8±3.7	19.5±5.7	20.2±2.1	ns	*	ns
$\Psi\pi_{sat}$ glucose (MPa)	-0.03±0.01 b	-0.05±0.01 b	-0.12±0.03 a	-0.10±0.02 a	ns	***	*
$\Psi\pi_{sat}$ fructose (MPa)	-0.06±0.02	-0.09±0.02	-0.10±0.03	-0.11±0.01	ns	*	ns
	Xylem						
Glucose (mg g^{-1}DW)	2.5±0.7	2.9±1.0	3.4±0.7	3.0±0.8	ns	ns	ns
Fructose (mg g^{-1}DW)	3.8±1.3	3.2±1.2	2.9±0.9	2.7±0.4	ns	ns	ns
$\Psi\pi_{sat}$ glucose (MPa)	-0.02±0.00	-0.02±0.01	-0.02±0.00	-0.02±0.00	ns	ns	ns
$\Psi\pi_{sat}$ fructose (MPa)	-0.03±0.01	-0.02±0.01	-0.02±0.00	-0.01±0.00	ns	*	ns

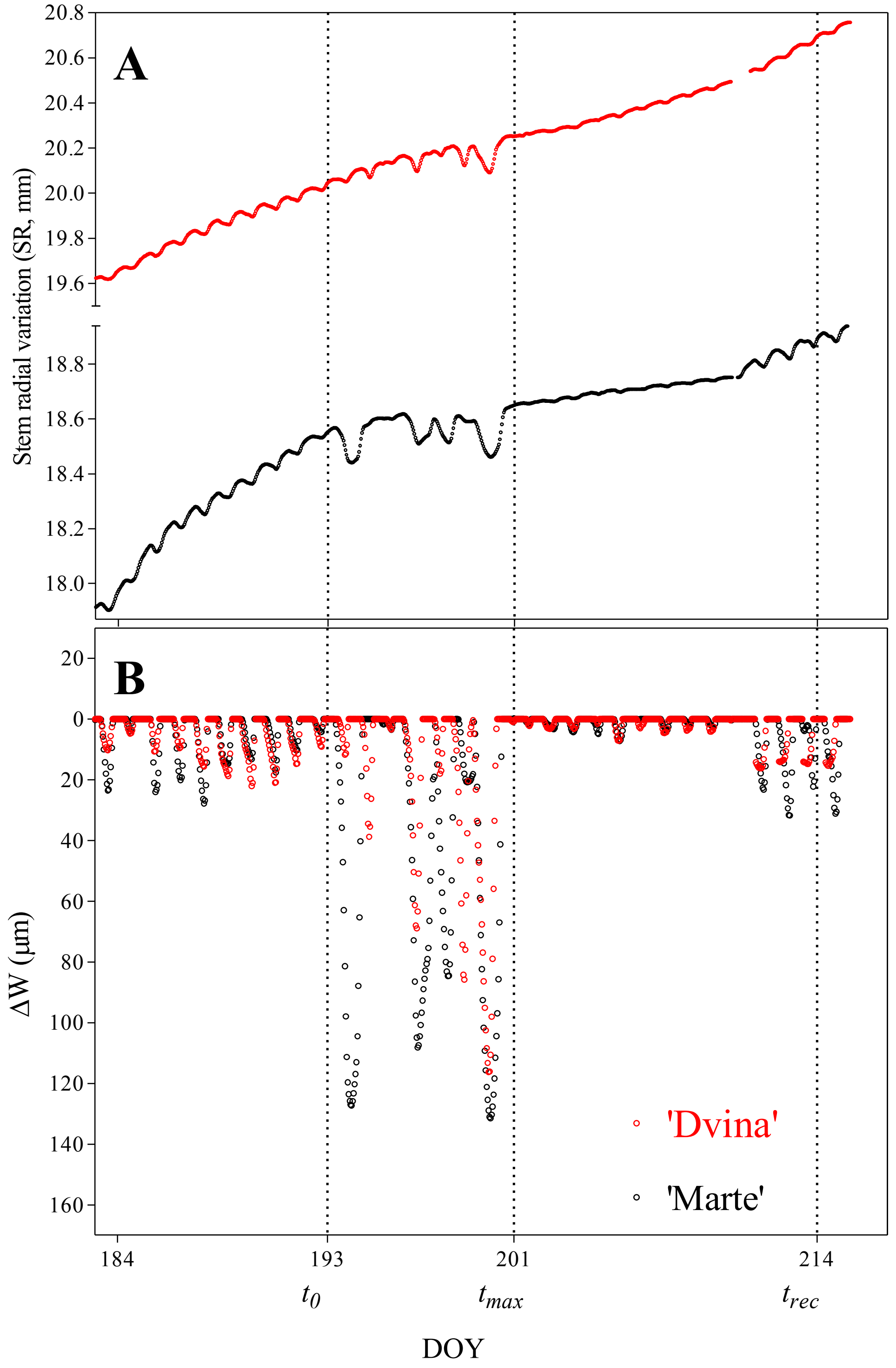

Figure S1. Stem radial variation SR (A) and instantaneous stem water deficit ΔW (B), extracted by de-trending the original growth data collected from the dendrometers with the help of piecewise linear regression, in *P. deltoides* 'Dvina' and *P. alba* 'Marte' during the experiment at t_0 (0 d), after 8 d of withholding water (t_{max}), and 13 d after resumption of irrigation (t_{rec}). Data in the figure corresponds to a single representative plant for each genotype.

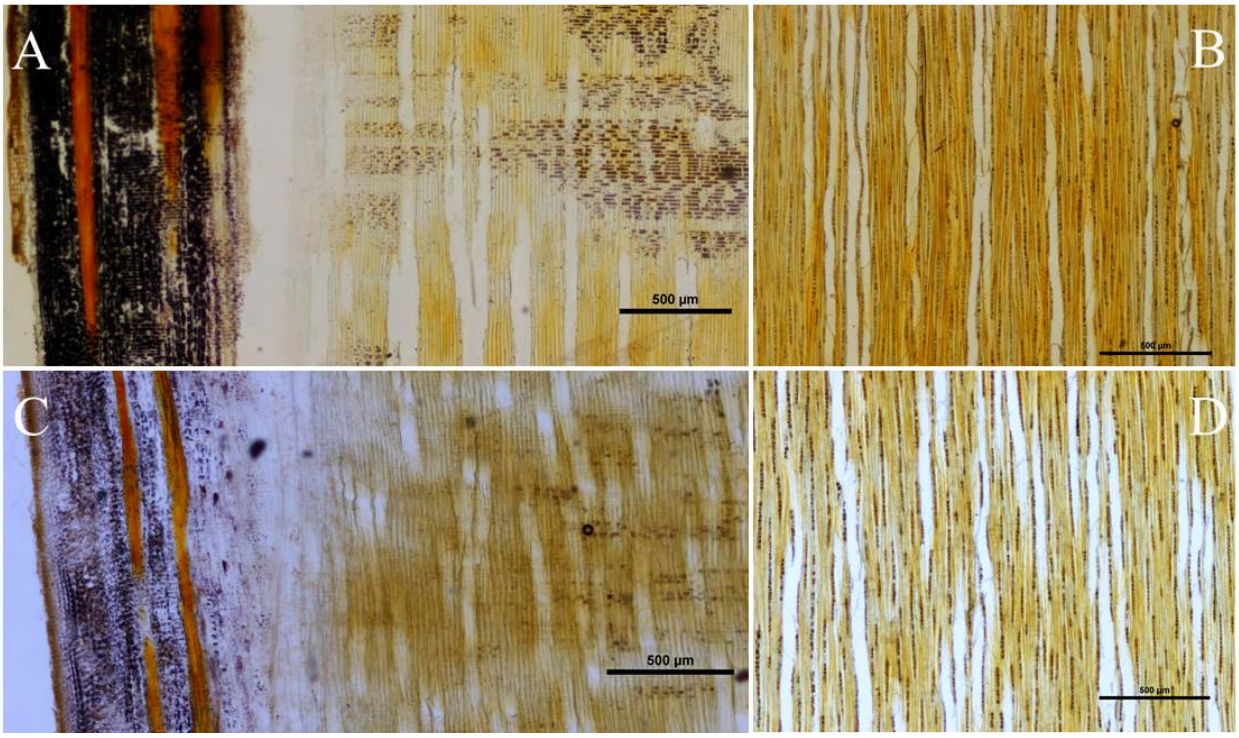

Figure S2. Starch localizations using Lugol's solution in the cambial region (A and C) and xylem (B and D). Stem transversal sections of 'Dvina' and 'Marte' plants sampled at t_0 (A and C, respectively). Stem tangential sections of 'Dvina' plants sampled at t_0 (B) and t_{rec} (D). Starch is stained in dark purple.

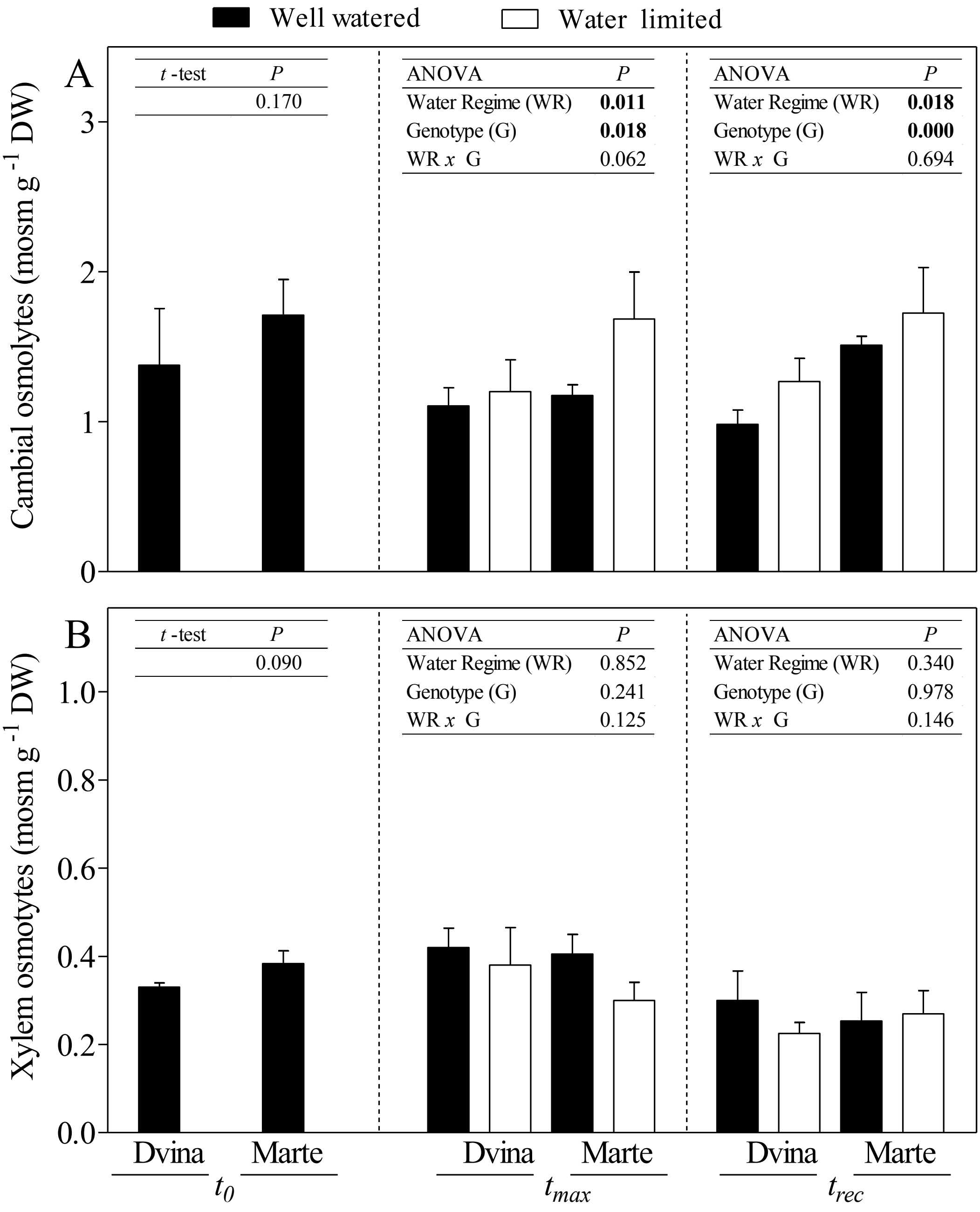

Figure S3. Osmotically active molecules in the cambial region (A) and xylem (B) of *P. deltoides* 'Dvina' and *P. alba* 'Marte' at t_0 (0 d), after 8 d of withholding water (t_{max}), and 13 d after resumption of irrigation (t_{rec}). The bars represent the mean of four biological replicates + SD. Data were analysed with *t*-test (t_0) or two-way ANOVA (t_{max} and t_{rec}). Significant differences are reported in bold print.

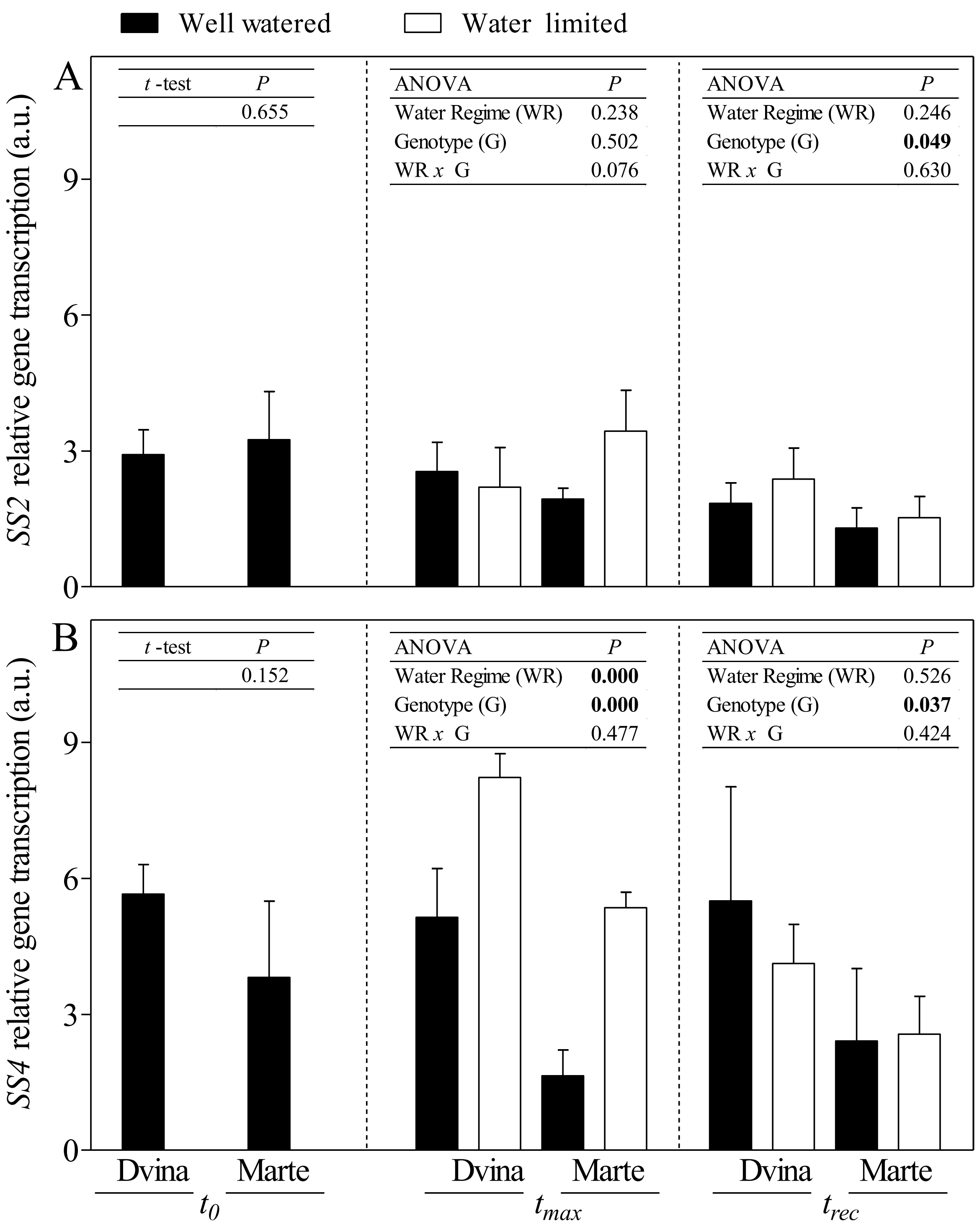

Figure S4. mRNA accumulations of *SS2* (A) and *SS4* (B) genes in the cambial region of *P. deltoides* 'Dvina' and *P. alba* 'Marte' at t_0 (0 d), after 8 d of withholding water (t_{max}), and 13 d after resumption of irrigation (t_{rec}). The bars represent the mean of three biological replicates + SD. Data were analysed with *t*-test (t_0) or two-way ANOVA (t_{max} and t_{rec}). Significant differences are reported in bold print.

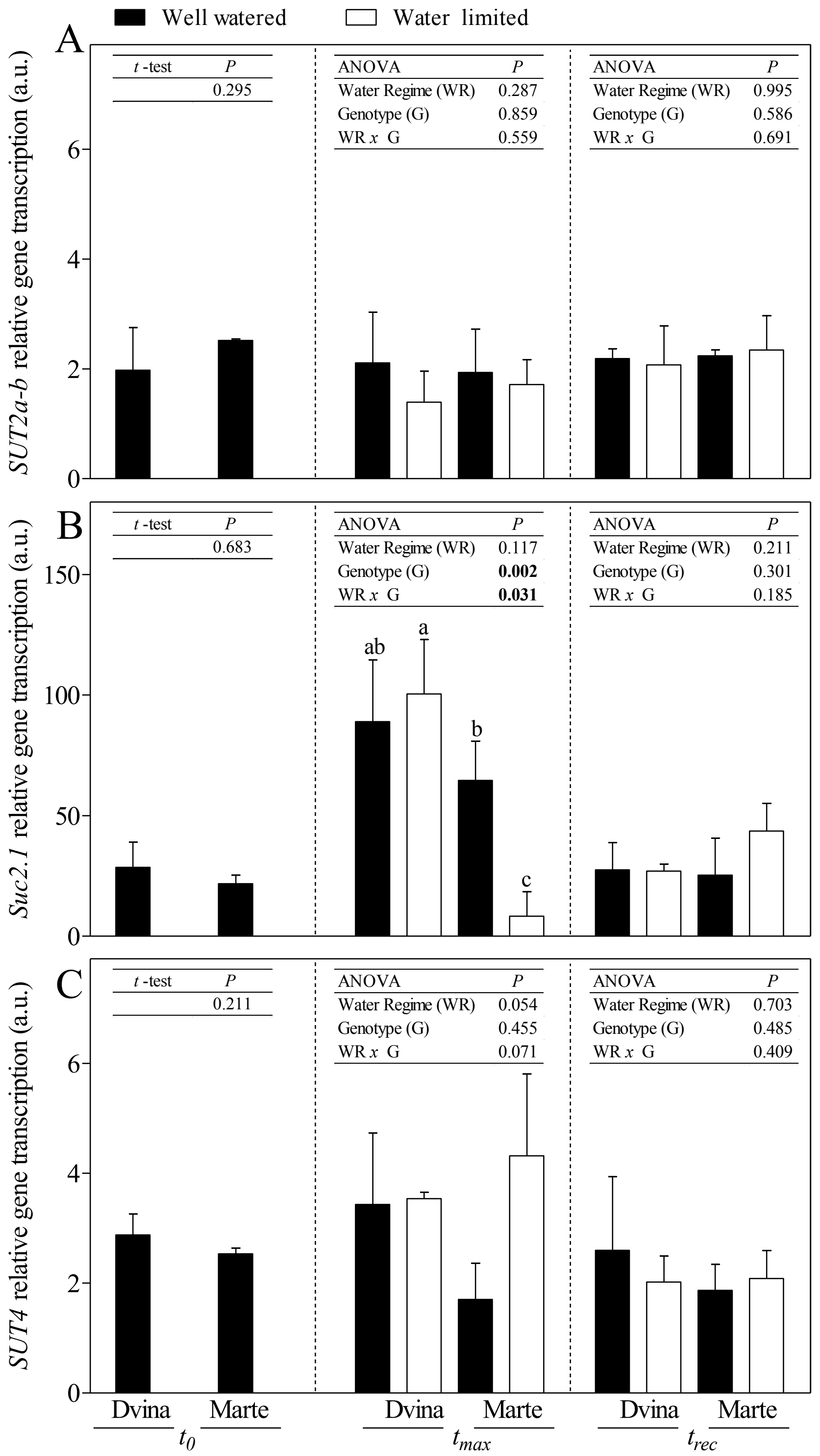

Figure S5. mRNA accumulations of *SUT2* (A), *Suc2.1* (B), and *SUT4* (C) genes in the cambial region of *P. deltoides* 'Dvina' and *P. alba* 'Marte' at t_0 (0 d), after 8 d of withholding water (t_{max}), and 13 d after resumption of irrigation (t_{rec}). The bars represent the mean of three biological replicates + SD. Data were analysed with *t*-test (t_0) or two-way ANOVA (t_{max} and t_{rec}). Significant differences are reported in bold print. The means were compared using Fisher's LSD test. Different letters indicate significant differences ($P < 0.05$).
